# Preparation of Ce-Doped Gd_3_(Al, Ga)_5_O_12_ Nanopowders via Microwave-Assisted Homogenization Precipitation for Transparent Ceramic Scintillators

**DOI:** 10.3390/ma17061258

**Published:** 2024-03-08

**Authors:** Min Liu, Yansen Zhang, Song Hu, Guohong Zhou, Xianpeng Qin, Shiwei Wang

**Affiliations:** 1 School of Materials Science & Engineering, Shanghai Institute of Technology, Shanghai 201418, China; liumin1106@ustc.edu.cn (M.L.); 206081160@mail.sit.edu.cn (Y.Z.); 2 Shanghai Institute of Ceramics, Chinese Academy of Sciences, Shanghai 201899, China; xpqin@mail.sic.ac.cn (X.Q.); swwang51@mail.sic.ac.cn (S.W.)

**Keywords:** Ce: GGAG, microwave-assisted homogenization precipitation, transparent ceramic, scintillation

## Abstract

Ce-doped gadolinium gallium aluminum oxide (Ce: GGAG) precursors were first prepared by the microwave-assisted homogeneous precipitation method (MAHP). Thermal gravity-differential thermal analysis (TG-DTA), X-ray diffraction (XRD), specific surface area analysis (BET) and field emission scanning electron microscopy (FE-SEM) were employed to investigate the crystal structure, phase evolution and morphologies of the Ce: GGAG precursors and powders. The influence of Ga ion concentration in the salt solution on the properties of Ce: GGAG powders was investigated. All the precursors were transformed into single-phase GGAG after being calcined at 950 °C in a furnace for 3 h. Monodispersed Ce: GGAG powders were obtained as the Ga ion concentration was lower than 0.06 mol/L. Single-phase and dense Ce: GGAG ceramics were obtained after sintering at 1600 °C in a flowing oxygen atmosphere for 10 h. Specifically, the Ce: GGAG ceramic reached its maximum density of ~6.68 g/cm^3^, which was close to its theoretical density of 6.70 g/cm^3^, and exhibited the highest optical transmittance of 65.2% at 800 nm after hot isostatic pressing sintering (HIP) as the Ga ion concentration was 0.02 mol/L. The decay time and light yield of the GGAG ceramic were 35 ns and 35,000 ± 1250 ph/MeV, respectively, suggesting that Ce: GGAG ceramics prepared using MAHP-synthesized nanopowders are promising for scintillation applications.

## 1. Introduction

Scintillators are materials converting high-energy radiation into visible photons, which are critical materials for scintillation detectors and have been widely applied in the fields of security inspection, high-energy physics, nuclear medicine and geological exploration [1]. In such applications, scintillators are generally required to possess merits including high light yield, high effective atomic number, high transmittance, fast decay time and low afterglow level. Typically, scintillators such as Bi_4_Ge_3_O_12_, Gd_2_O_2_S: Pr, Lu_2_SiO_5_: Ce, Gd_2_SiO_5_: Ce, CsI: Tl and Ce: Y_3_Al_5_O_12_ have been widely explored and applied [2,3,4,5,6]. Among the explored materials, single-crystal-based scintillators are most famously used for X-ray computed tomography (X-CT) systems owing to their high optical property, high light output, and high energy resolution [7]. However, the cost of single crystals remains ultra-high and the production cycle is ultra-long due to their harsh synthesis conditions including high temperature, low growth speed and the high cost of the crucible for crystal growth [8]. By contrast, ceramic-based inorganic scintillators are attracting increasing attention due to the relatively short fabrication cycle and friendly preparation conditions, and it is more flexible to prepare ceramic scintillators of a large size. Ceramic scintillators such as Eu: (Y, Gd)_2_O_3_ and Gd_2_O_2_S: Tb(Ce) have been pioneered since the late 1980s [9,10], which opened up the application of ceramic scintillators in the field of imaging. Nevertheless, ceramic scintillators had limited applications because of their opaque properties, and they had to be applied in a thin plate form [11]. Fortunately, the technological progress in shaping and sintering has developed fast, making it possible to fabricate transparent ceramics with high optical quality. Moreover, transparent ceramics are attractive for their high physical–chemical stabilities, unique distortion structures and the flexibility of doping or co-doping with high-level rare-earth ions [12].

In the past few decades, a growing category of ceramic scintillators such as lutetium (yttrium) aluminum garnet ceramics, fluoride ceramics, silicate ceramics and sesquioxide ceramics have been widely studied [13,14,15,16]. Part of the ceramics even exhibited superior scintillation properties compared to those of single crystals with the same chemical composition [17]. However, the applications of most of the above transparent ceramics were limited due to deficiencies in attenuation time or light output, making it highly necessary to seek new ceramic scintillators. Notably, the band engineering of Lu_3_Al_5_O_12_ by substituting Lu^3+^ and Al^3+^ with Gd^3+^ and Ga^3+^ ions, respectively, to form Gd_3_(Al, Ga)_5_O_12_ (GGAG) transparent ceramics effectively keeps the advantages of conventional garnet ceramics, such as the feasibility of obtaining high optical properties, and the dopants can be contained in larger quantities. Ce-doped GGAG ceramic scintillators have been reported to be compelling due to the high light output as well as low primary decay time for Ce^3+^ activator ions [18]. Researchers at Lawrence Livermore National Laboratory have reported a 5 in^3^ Ce-doped GYGAG transparent ceramic using the fabricated ceramic scintillator for lens-coupled radiographic imaging applications [19]. However, they have not decoded the details of the fabrication procedure or the in-line transmittance of the as-fabricated transparent ceramic. 

To the best of our knowledge, only a few studies have reported addressing transparent Ce: GGAG ceramics, mainly due to the component segregation and easy volatilization of the Ga_2_O_3_ component. Specifically, the starting powders should be tightly controlled, and powders with high purity, superior cationic dispersion, high reactivity and a high degree of crystal perfection are required for high-quality Ce: GGAG transparent ceramic scintillators. Currently, two methods are usually adopted to synthesize Ce: GGAG powders, i.e., the solid-state reaction and wet chemical methods. For the solid-state reaction method, a high temperature (>1300 °C) and long calcination time (>10 h) are usually required to obtain single-phase Ce: GGAG, which easily leads to the volatilization and decomposition of Ga_2_O_3_ and the formation of impurities in the powders. Consequently, the as-fabricated Ce: GGAG ceramics show an opaque appearance [20]. Only a few research groups have successfully fabricated highly transparent Ce: GGAG ceramics via the solid-state reaction method. Compared with the solid-state reaction method, wet chemical synthesis methods could realize the uniform mixing of ions, which is beneficial for obtaining single-phase Ce: GGAG using a relatively low calcining temperature. Jiang and co-workers [21,22] have successfully prepared well-dispersed and nano-sized Ce: GGAG powders by a co-precipitation (CC) method and an ultrasonic-assisted chemical coprecipitation (UACC) method, respectively. The researchers found that the ceramics fabricated from the UACC method exhibit higher density than those fabricated from the CC powders, which might be due to the larger specific surface area and higher uniformity of the UACC powders. Unfortunately, the optical and scintillation properties of the ceramics have not been reported. Although the UACC method has promoted the properties of nano-sized powders, the powders might still suffer deficiencies including nonuniformity of components because of uneven temperatures and a long aging time. The microwave-assisted homogeneous precipitation method (MAHP) is a rapid, facile and reproducible method for preparing monodispersed nano-sized powders due to its fast and uniform microwave heating characteristics. Such a method has been applied to synthesize homogeneous powders for various functional ceramics, including transparent garnet ceramics [23]. Therefore, it is extremely attractive for synthesizing Ce: GGAG ultrafine powders. To our knowledge, there are no reports on the Ce: GGAG powders prepared using the MAHP method.

In this manuscript, the MAHP method was applied in the preparation of Ce: GGAG nanopowders, and the influences of concentrations of metal ions in the salt solution on the properties of powders were explicitly investigated in detail. The dense ceramics fabricated from the as-prepared powders were evaluated, and the application potentials of the transparent ceramics in scintillation detectors have been preliminarily discussed.

## 2. Experimental 

### 2.1. Materials

Gadolinium chloride hexahydrate (GdCl_3_·6H_2_O) (>99.9% purity, Shanghai Aladdin Biochemical Technology Co., LTD.), anhydrous gallium chloride (GaCl_3_) (>99.99% purity, Shanghai Aladdin Biochemical Technology Co., Ltd., Shanghai, China), Cerium(III) chloride heptahydrate(CeCl_3_·7H_2_O) (>99.99% purity, Shanghai Aladdin Biochemical Technology Co., LTD.) and aluminum chloride hexahydrate (AlCl_3_·6H_2_O) (>99.99% purity, Shanghai Aladdin Biochemical Technology Co., Ltd.) were adopted as the raw materials. Urea (CO (NH)_2_)_2_, A.R.) and ammonium sulfate ((NH_4_)_2_SO_4_, A.R., Shanghai Lingfeng Chemical Reagent Co., Ltd., Shanghai, China) were used as the precipitant and the surfactant, respectively. 

### 2.2. Preparation of Ce: GGAG Nanopowders

**Figure 1:**
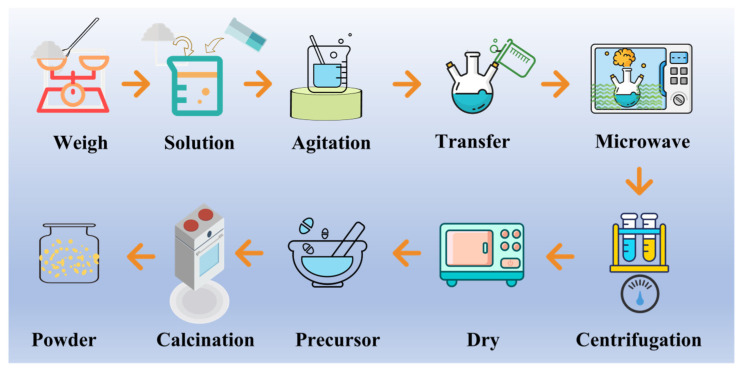
Schematic diagram of Ce: GGAG nanopowders prepared by the MAHP method.

The aqueous solutions were prepared by dissolving GdCl_3_·6H_2_O, GaCl_3,_ AlCl_3_·6H_2_O, and CeCl_3_·7H_2_O in deionized water, and the solutions were mixed according to the stoichiometric ratio of {[Gd^3+^] + [Ce^3+^]}:{[Ga^3+^] + [Al^3+^]} = 3:5, wherein the doping concentration of Ce^3+^ was determined to be 0.33 at.%. Traces of (NH_4_)_2_SO_4_ were dissolved in the solutions. The optimal molar ratio of urea to the total metal ions ([U]/[M]) was determined to be 35:1 by adding an appropriate amount of urea into the solution. The solutions with various Ga ion concentrations (i.e., 0.005, 0.02, 0.04, 0.06, 0.08, and 0.1 mol/L, designated as 0.005, 0.02, 0.04, 0.06, 0.08, 0.1 nanopowder, respectively) were prepared by diluting the salt solution with deionized water. The homogeneous synthesis was carried out in a microwave-ultrasonic reaction system (microwave frequency 2.45 GHz, Xian-ou Instrument Co., Ltd., Nanjing, China) until visible turbidity appeared. Detailed microwave parameters were described in the results and discussion sections. Furthermore, the obtained suspension was isolated by centrifugation and washed three times with deionized water and ethanol. Afterward, the products were dried at 60 °C for 24 h, and nanopowders were obtained after being calcined at 850–1100 °C. The schematic diagram for synthesizing Ce: GGAG nanopowders is shown in Figure 1.

### 2.3. Preparation of Ce: GGAG Ceramics

The synthesized Ce: GGAG nanopowders were compressed uniaxially in a steel die and cold isostatically pressed at 200 MPa to obtain compacted green bodies. The Ce: GGAG ceramics were prepared after being sintered at 1600 °C for 10 h in an oxygen atmosphere according to various nanopowders, designated as 0.005, 0.02, 0.04, 0.06, 0.08, 0.1 ceramic, respectively. To achieve higher density and optical transmittance, the obtained ceramic samples were hot isostatically pressed (HIP) under 200 MPa of argon gas pressure at 1480 °C for 2 h. Finally, all the samples underwent a mirror-polishing process on both faces into 1 mm in thickness and thermally etched at 1300 °C for 1 h for grain size measurement and scintillation performance characterization. Before testing, the ceramic samples and the BGO single crystal (produced by Shanghai Institute of Ceramics, CAS, Shanghai, China) were double-face polished to a size of 15 mm × 15 mm × 1 mm. 

### 2.4. Characterizations

A thermogravimetric and differential thermal analysis (TG-DTA) of the dried precursor was conducted on a thermal analysis instrument (TG/DTA, Model STA449C, Netzsch, Germany) from room temperature to 1200 °C at a heating rate of 10 °C min^−1^ while air flowed. The phase compositions of the nanopowders and ceramics were determined by powder X-ray diffraction (XRD) employing Cu Kα radiation (λ = 1.5405 Å). The morphologies of nanopowders were observed by field emission scanning electron microscopy (FE-SEM, Model Carl Zeiss 1550, Carl Zeiss Electron Co., Jena, Germany). Surface morphologies of the ceramics were observed by scanning electron microscopy (SEM) (JSM-6390LA, JEOL, Tokyo, Japan) under a working voltage of 20 kV. The BET method determined the specific surface area of the calcined GGAG powders. The optical transmittance was measured by a UV-VIS-NIR spectrophotometer (V770, JASCO, Tokyo, Japan). The Archimedes principle was used to measure the density of sintered ceramics. The scintillation decay time was measured by pulsed X-ray excitation with a pulse width of 2 ns. To determine the scintillation light yield (LY), pulse height spectra measurements were carried out by using a multichannel spectrometer (DigiBASE, ORTEC, Oak Ridge, TN, USA). A collimated gamma-ray source (^137^Cs) was used to excite the sample, and a standard evaluation procedure was applied (calibration by a single photoelectron peak position and using the PMT quantum efficiency (QE) spectral dependence to obtain the ph/MeV value). 

## 3. Results and Discussion

A two-step procedure was developed to control the nucleation and growth of nano-powders during the process of synthesizing Ce: GGAG nanopowders by microwave-assisted homogeneous precipitation. Initially, a solution with a certain Ga ion concentration was heated at a microwave power of 1200 W for 4 min. Subsequently, the microwave power was reduced to 200 W and maintained for 30 min until the precipitates were obtained; the reactions happened as follows:

CO(NH_2_)_2_ + 2H_2_O ⇌ (NH_4_)_2_CO_3_ (1)

(NH_4_)_2_CO_3_ + H_2_O ⇌ NH_4_HCO_3_ + NH_3_·H_2_O (2)

HCO_3_^−^ + H_2_O ⇌ H_2_CO_3_ + OH^−^ (3)

NH_3_·H_2_O ⇌ NH_4_ ^+^ + OH^−^ (4)

OH^−^ + CO_3_^2−^ + Gd^3+^ + Ga^3+^ + Al^3+^ → Gd_3_(Al, Ga)_5_(OH)_x_(CO_3_)_y_·nH_2_O ↓ (5)

**Figure 2:**
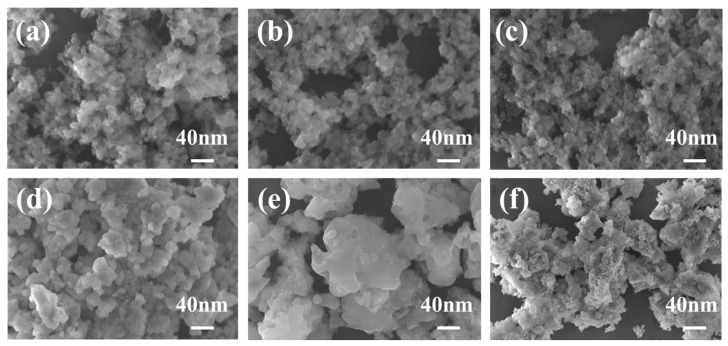
FE-SEM images of the typical precursors synthesized via the MAHP method with Ga ion concentrations of (**a**) 0.005, (**b**) 0.02, (**c**) 0.04, (**d**) 0.06, (**e**) 0.08 and (**f**) 0.1 mol/L, respectively.

The resulting precipitates were then subjected to washing, drying, grinding and sieving; precursors with nano-sized particles could be obtained. Apart from the particle size, the morphology of the particles is also critical for the densification of ceramics. To regulate the particle size and morphology of the precursors and successfully obtain single-phase Ce: GGAG powders with monodisperse characteristics, solutions with varying Ga ion concentrations ranging from 0.005 to 0.1 mol/L were prepared. Influences of Ga ion concentration in the salt solution on microstructures of the precursors could be observed in Figure 2. As the Ga ion concentration was as low as 0.005, 0.02 and 0.04 mol/L, particle sizes of the precursors were ultrafine; more significantly, they were well dispersed. The particles started to aggregate with the increase in Ga ion concentration up to 0.06 mol/L or even higher. Therefore, the low metal ion concentration contributed to monodispersed precursors. Considering the low production of 0.005 mol/L salt solutions, the precursors prepared from salt solutions with a Ga ion concentration of 0.02 mol/L were further investigated. 

**Figure 3:**
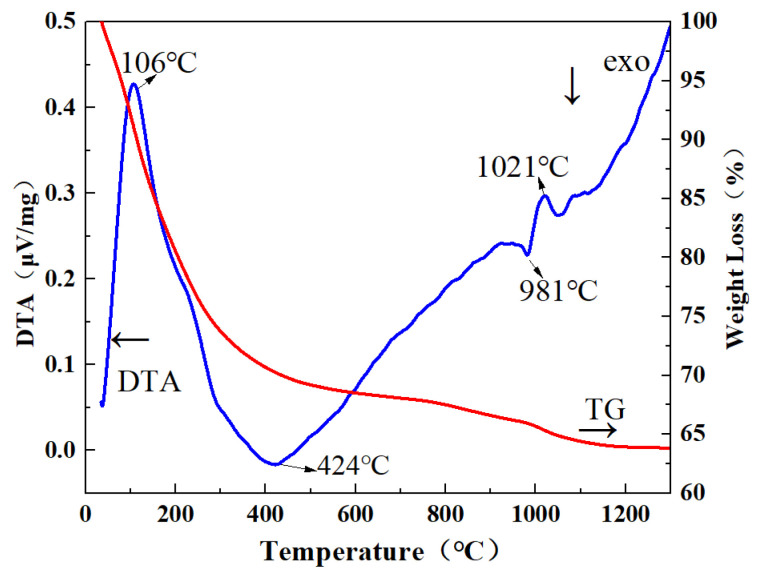
Thermogravimetry/differential thermal analysis traces showing the decomposition process of the 0.02 precursors synthesized via the MAHP method.

TG/DTA was performed on the 0.02 precursor, as shown in Figure 3. The TG curve showed a total mass loss of 36.17 wt.% as the calcinating temperature increased from room temperature to 1200 °C, which was ascribed to the dehydration process and decomposition of NH_4_^+^ and CO_3_^2−^, as well as residual NO_3_^−^ and SO_4_^2−^. It can be clearly seen from the DTA curve that a prominent exothermic peak appeared at 981 °C, which might correspond to a phase transition to GGAG.

**Figure 4:**
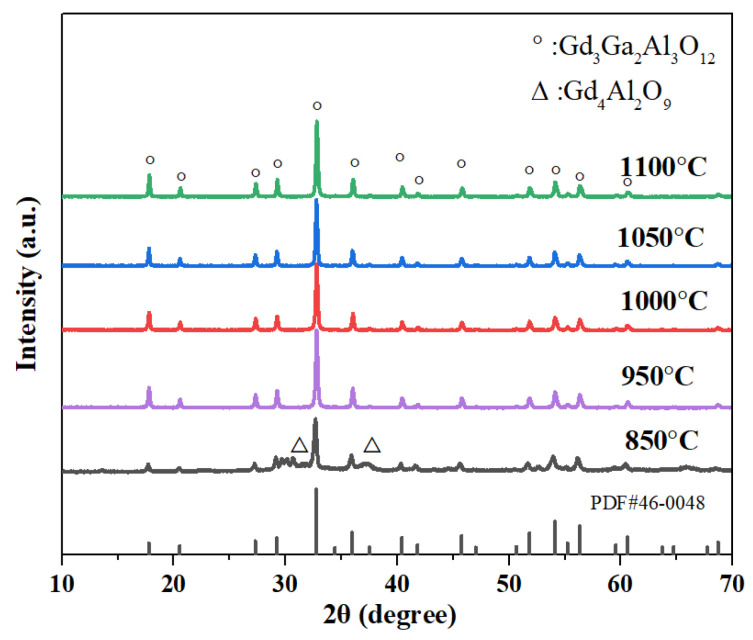
XRD patterns of 0.02 nanopowders calcined at different temperatures ranging from 850 to 1100 °C.

The 0.02 precursors were calcined at various temperatures ranging from approximately 850 to 1100 °C, respectively, according to the TG/DTA curves. Figure 4 displays the XRD patterns of the powders. The majority of the characteristic peaks of GGAG and weak peaks of the Gd_4_Al_2_O_9_ phase were observed between 30° and 32° (2*θ*) as calcined at 850 °C, indicating that higher temperatures are required to achieve single-phase GGAG powders. As the temperature increased to 950°C, the diffraction peaks of Gd_4_Al_2_O_9_ phase disappeared, only GGAG garnet structured characteristic peaks (PDF#46-0448) could be observed. As the calcinating temperature gradually increased to 1100 °C, no impurities were produced, and the relative intensities of the main diffraction peak representing miller indices (420) towards other peaks increased, indicating the well growth of GGAG crystallites. The lattice constant of the 0.02 nanopowders calculated from XRD data using MDI Jade 6.5 software was approximately 12.25 Å, further indicating that pure GGAG nanopowders were obtained after being calcined at 950 °C.

**Figure 5:**
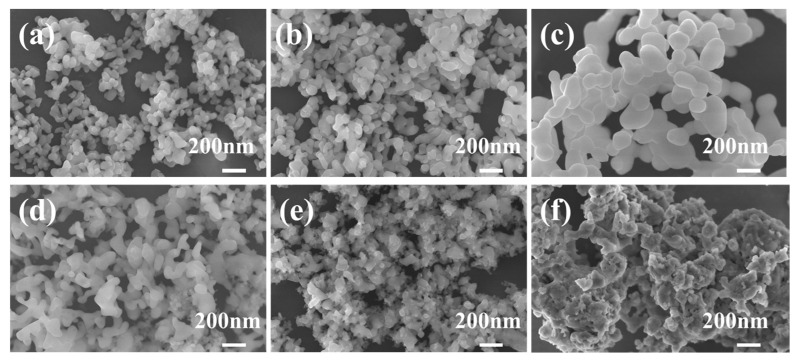
FE-SEM images of the 0.02 nanopowders calcined at (**a**) 950 °C, (**b**) 1050 °C, and (**c**) 1100 °C, respectively. (**d**–**f**) FE-SEM images of the 0.06, 0.08 and 0.10 nanopowders calcined at 950 °C.

Figure 5a–c display FE-SEM images of the 0.02 nanopowders calcined at different temperatures. The images reveal that the 0.02 nanopowders exhibit excellent dispersibility and uniformity of shape, even after being calcined at high temperatures. The particle size of the nanopowders was estimated to be about 50 nm as the calcinating temperature was 950 °C. With higher calcinating temperatures, the particles proliferated, but the dispersibility and uniformity changed little. At the same time, the 0.005, 0.04, 0.06, 0.08, and 0.10 nanopowders were also prepared by calcinating the corresponding precursors at 950 °C for comparison. FE-SEM images of the 0.06, 0.08 and 0.10 nanopowders displayed in Figure 5d–f indicated that the nanopowders possessed irregular shapes and noticeable agglomerates as salt solutions with high metal ion concentrations were used, the agglomerates become more pronounced for the 0.10 precursors. Further, the specific surface areas of the 0.02, 0.06, and 0.10 nanopowders calcined at 950 °C were measured to be 12.5, 11.7, and 5.2 m^2^/g, respectively. The average particle size was estimated to be 53, 57, and 82 nm, respectively, consistent with the FE-SEM results. Increased Ga ion concentration higher than 0.02 mol/L led to larger particle sizes and a higher degree of agglomeration for precursors, and the dehydration during the calcination process could further result in the formation of oxygen bridges and hard agglomerates. This might be the reason for the low BET surface area of high-concentration samples (e.g., 0.06 and 0.1 mol/L). It is widely acknowledged that achieving ceramics with high relative density and excellent optical transmittance requires a high sintering activity of powders with proper specific surface area, uniform morphology, and pure crystal phase. The hard agglomeration of nanopowders was significantly detrimental to the densification of ceramics. Other typical wet chemistry methods including homogeneous coprecipitation and sol–gel method have also been successfully adopted for the preparation of single-phase garnet nanopowders. However, the morphologies and particle sizes could not be easily tailored since hours or even days of aging time or chelation and crosslink processes are generously required [24,25]. On the contrary, the morphology and dispersibility characteristics can be significantly regulated by adjusting the concentration of Ga ion via the currently studied MAHP method. 

**Figure 6:**
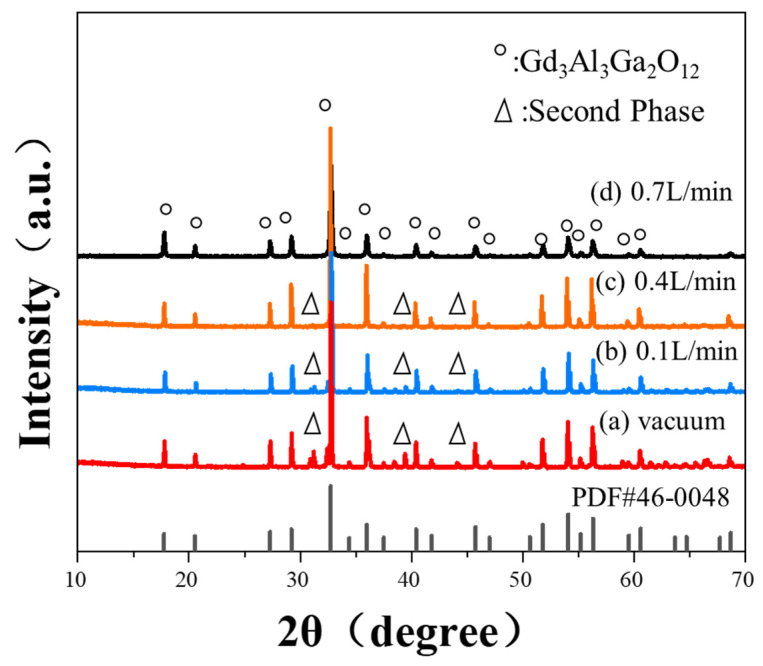
XRD patterns of the 0.02 ceramics pre-sintered at 1600 °C and held for 10 h under different atmospheres: (a) vacuum, oxygen flow of (b) 0.1, (c) 0.4 and (d) 0.7 L/min.

The densification of GGAG ceramics is influenced by the sintering temperature, as supported by literature and the GGAG compound phase diagram [26]. Based on this information, a pre-sintering temperature of 1600 °C under vacuum was first chosen to promote the removal of pores on the grain boundaries. However, the vacuum pre-sintering atmosphere led to the decomposition of GGAG, as shown in Figure 6a, the secondary phase composed of Gd_4_Al_2_O_9_ and (Al, Ga)_2_O_3_ appeared in the 0,02 ceramic. This might be due to the evaporation of Ga-O at high temperatures with low oxygen partial pressure [27]. Sintering additives such as TEOS, MgO, and ZrO_2_ have usually been applied to lower the sintering temperature for fully densified GGAG ceramics [28,29,30]. However, using sintering aids simultaneously introduces impurities in the ceramics, which would affect the scintillation performances of the materials. For instance, the co-doping of alkaline earth metals ions (e.g., Mg^2+^ or Ca^2+^) would lead to a higher proportion of Ce^4+^ due to charge compensation. In addition, relatively high impurities on the grain boundaries would lead to the degradation of the resolution of the imaging applications. Herein, pre-sintering in an oxygen-rich atmosphere was adopted in the current work to inhibit the Ga evaporation effectively. As can be observed in Figure 6b–d, relative intensities of the diffraction peaks representing impurities decreased gradually with the increase in oxygen flow. As 0.7 L/min oxygen flow was applied, the pure phase of GGAG could be obtained, demonstrating that higher oxygen partial pressure could effectively inhibit the evaporation of Ga and the decomposition of GGAG.

**Figure 7:**
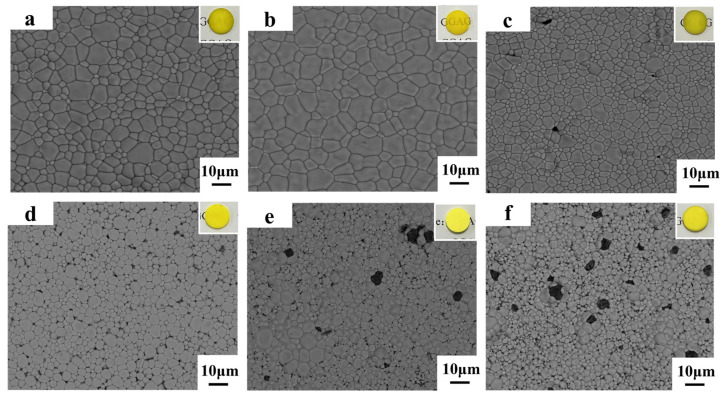
The surface morphologies of (**a**) 0.005, (**b**) 0.02, (**c**) 0.04, (**d**) 0.06, (**e**) 0.08 and (**f**) 0.1 ceramics sintered at 1600 °C for 10 h under 0.7 mol/L oxygen flow.

**Figure 8:**
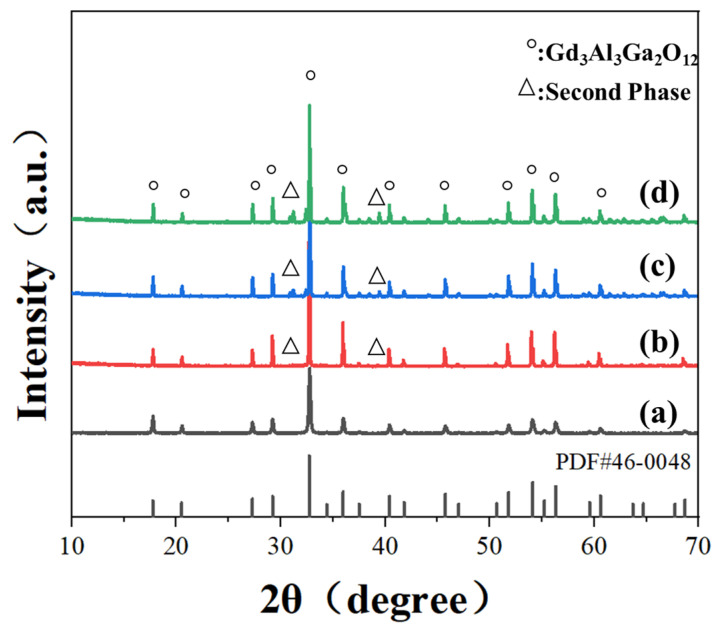
XRD patterns of the pre-sintered ceramics from (a) 0.04, (b) 0.06, (c) 0.8, (d) 0.10 nanopowders.

Figure 7b shows the surface morphologies and appearances of the pre-sintered 0.02 ceramic. It can be observed that words below the ceramic sample could be obscurely read, indicating that the impurities and pores inside the ceramic were removed to a great extent. The grain size was estimated to be about 10 μm from the FE-SEM image, and few micropores were observed. For comparison, the 0.005, 0.04, 0.06, 0.08, and 0.1 ceramics were also prepared at 1600 °C for 10 h under the oxygen-rich atmosphere. The 0.02 ceramic has an average grain size that is larger than those of other pre-sintered ceramics. As the Ga ion concentration increased, the number of pores and secondary phases gradually increased as well, as depicted in Figure 7c–f, suggesting that the powders prepared from a relatively low Ga ion concentration significantly contributed to the high densification and uniform grain growth and higher metal ion concentration might be detrimental to the densification. Specifically, the 0.06, 0.08, and 0.10 ceramics exhibited large-sized pores, abnormal grain growth, and secondary phases. This might be ascribed to the hard agglomeration of the powders, as can be observed in Figure 5. XRD profiles were further conducted for the pre-sintered ceramics, as shown in Figure 8. It was worth noting that the 0.04 ceramics exhibited a pure GGAG phase after sintering at 1600 °C in a flowing oxygen atmosphere for 10 h. However, diffraction peaks representing secondary phases were observed in the 0.06, 0.08 and 0.10 ceramics. The secondary phases probably inhibited the migration of grain boundaries and pores, leading to smaller grain sizes and pores, as shown in Figure 7c–f. The MAHP method, coupled with a low Ga ion concentration, has been identified as an effective solution for promoting the densification of single-phase Ce: GGAG ceramics.

**Figure 9:**
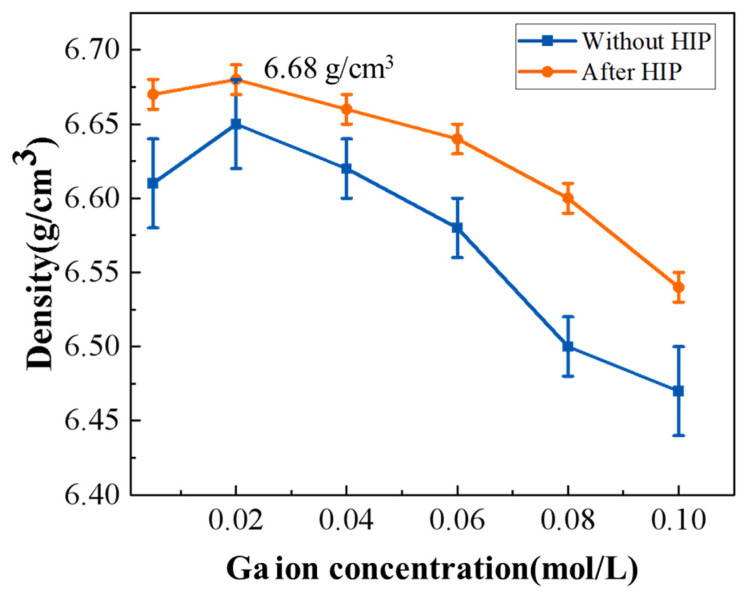
Densities of Ce: GGAG ceramics sintered from 0.005, 0.02, 0.04, 0.06, 0.08, 0.1 nanopowders.

**Figure 10:**
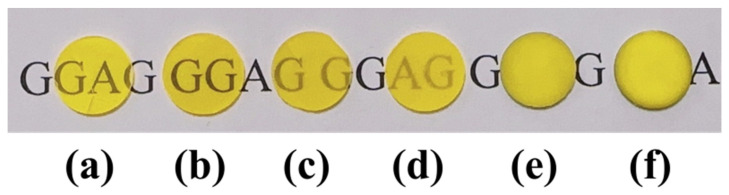
Photographs of Ce: GGAG ceramics after HIP: (**a**) 0.005, (**b**) 0.02, (**c**) 0.04, (**d**) 0.06, (**e**) 0.08 and (**f**) 0.1 ceramic.

Figure 9 presents the densities for the pre-sintered 0.005, 0.02, 0.04, 0.06, 0.08, and 0.1 ceramics before (blue dots) and after (orange dots) the HIP procedure. It was clear that as the Ga ion concentration increased to higher than 0.02 mol/L, the densities gradually decreased, consistent with the SEM results shown in Figure 7. Furthermore, it can be deduced that the HIP procedure can significantly promote the densification of the pre-sintered ceramics. The density of the 0.02 ceramic reached a maximum of 6.68 g/cm^3^ after HIP, which was close to the theoretical density of 6.70 g/cm^3^. The density was the same as that of the sample fabricated with the assistance of sintering additives [29], indicating that pre-sintering ceramics under an oxygen-rich atmosphere was promising for fully dense GGAG transparent ceramics. Figure 10 exhibited the appearance of the HIP-ed ceramics. It could be intuitively observed that after HIP, the transparency of the ceramics improved significantly. The 0.02 ceramic has the optimal optical quality (Figure 10b), and words under the ceramic could be read. 

**Figure 11:**
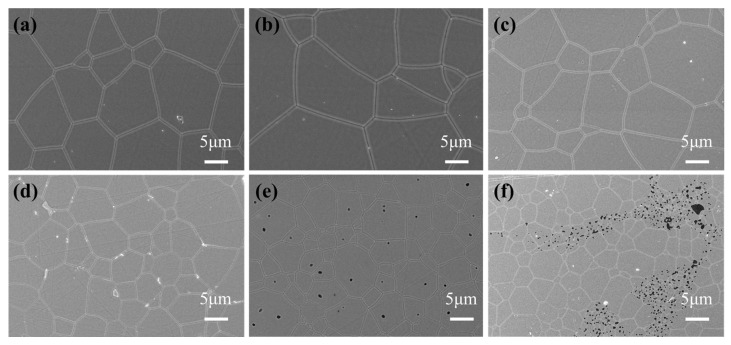
The surface morphologies of (**a**) 0.005, (**b**) 0.02, (**c**) 0.04, (**d**) 0.06, (**e**) 0.08 and (**f**) 0.10 ceramics after HIP.

**Figure 12:**
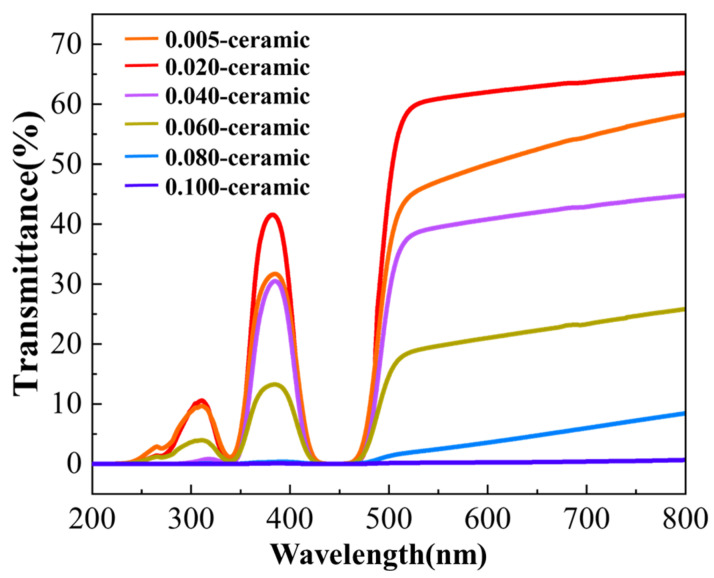
The optical transmittance curves of Ce: GGAG ceramics after HIP.

Figure 11 shows the surface morphologies of the Ce: GGAG ceramics after HIP. It can be found that after HIP, pores inside all the ceramics were extensively eliminated. However, large amounts of pores on the grain boundaries or inside the grains still existed in the 0.08 and 0.1 ceramics after HIP. The incident light passing through the heterogeneous interfaces will inevitably cause a continuous reflection and refraction of light, thus reducing its transmittance. Meanwhile, the grain sizes of these ceramic samples were calculated using linear intercepts based on the following equation:

(6) $$\bar{D}=1.56\bar{L}$$

where $\bar{D}$ is the average grain size of the ceramic and represents the average intercept length over a large number of grains as measured on a polished surface [31]. Based on this calculation, the average grain size is 15.4, 17.7, 12.4, 8.7, 5.2, and 3.4 μm, for the 0.005, 0.01, 0.02, 0.04, 0.06, 0.08, 0.10 ceramic, respectively. Being HIP-ed under the same temperature, the average grain size of the 0.02 ceramic is more significant than that of the 0.10 ceramic sample. This is consistent with the results shown in Figure 7, demonstrating that powders prepared from solutions with low cation concentrations have a more significant driving force on sintering and grain growth, and fewer pores inside the ceramic bodies would promote crystal growth. Figure 12 displays the in-line transmittance of the HIP-ed ceramics. It shows a similar phenomenon that the 0.02 ceramic has the highest in-line transmittance of 65.2% at 800 nm. However, as the Ga ion concentration increased to higher than 0.02 mol/L, the in-line transmittance decreased and sharply dropped to 25.8% at 800 nm for the 0.06 ceramic. From all the above results, it could be concluded that Ga ion concentration played an essential role in preparing transparent Ce: GGAG ceramics. Specifically, relatively low Ga ion concentrations are beneficial for preparing high-performance Ce: GGAG nanopowders and transparent ceramics.

The optical quality of the scintillators is significant for a wide array of imaging applications, including security inspection and medical diagnostics, especially those that require ultrahigh spatial resolution at a low radiation dose rate [19,32]. It is worth noting that there is still room for the improvement of the optical quality of the Ce: GGAG transparent ceramics by using nanopowders synthesized via MAHP since in-line transmittances of a Ce: GGAG transparent ceramic have already been reported to be as high as 78.6% in the visible wavelength range [28]. The optical performances of the Ce: GGAG transparent ceramics fabricated via a solid-state reactive method have been reviewed, as listed in Table 1 [29,33,34,35,36,37], from which, one can conclude that proper category and amount of sintering aids together with an optimized HIP process are critical for the full densification of GGAG transparent ceramics. Jiang and coworkers [38] reported a Ce: GGAG transparent ceramic with a relatively high transmittance of 68% by using powders prepared from a novel wet chemistry synthesis method called ultrasonic enhanced chemical co-precipitation method (UCC). The optical quality was slightly higher than that of the currently fabricated sample. The two-step sintering process, including HIP, was conducted to promote densification. However, no sintering aids were included. It could be deduced that by introducing sintering aids, the transmittance could be further improved. In addition, the authors [38] made a comparison between the conventional chemical co-precipitation method (TCC) and the novel UCC method for the preparation of GGAG nanopowders for transparent ceramics, it is worth affirming that monodispersed and single-phase powders are much more favorable for the densification and optical quality enhancement of the transparent ceramics. Therefore, the MAHP method used in the present work has demonstrated great potential for synthesizing GGAG powders for fabricating Ce: GGAG transparent ceramics. More sophisticated experimental parameters should be further considered in order to further improve the sintering property of the dense ceramics. Influences of the particle sizes, oxygen flow rate during pre-sintering, pre-sintering, and HIP technologic parameters, including sintering temperatures and soaking time, on the microstructure evolutions of the Ce: GGAG transparent ceramics should be further studied; the microstructure as well as the optical properties of the final GGAG transparent ceramics could probably be further improved without introducing sintering aids.

**Figure 13:**
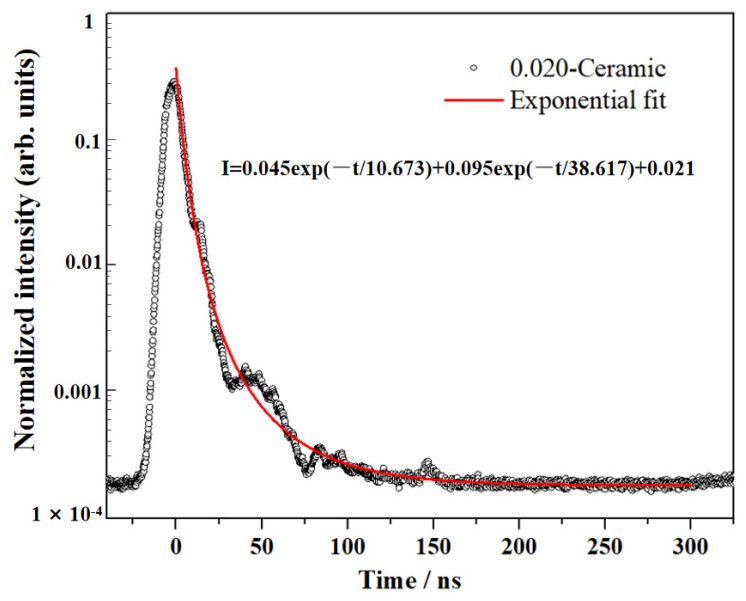
Scintillation decay curve of the 0.02 transparent ceramic.

The scintillation decay curve of the 0.02 transparent ceramic was presented in Figure 13, which could be approximated by a double exponential function *I* = *I*_0_ + A_1_exp(−t/τ_1_) + A_2_exp(−t/τ_2_). The result indicated that the as-prepared 0.02 transparent ceramic has a fast decay time of 35 ns, much faster than the Ce: GGAG single crystals and opaque ceramics [39]. It is widely acknowledged that scintillators with fast decay constants are crucial for scintillation counting applications, as they prevent ghosting caused by energy accumulation. The Ce: GGAG ceramic demonstrated fast scintillation decay, making it a potentially valuable scintillator for PET medicine and imaging applications.

**Figure 14:**
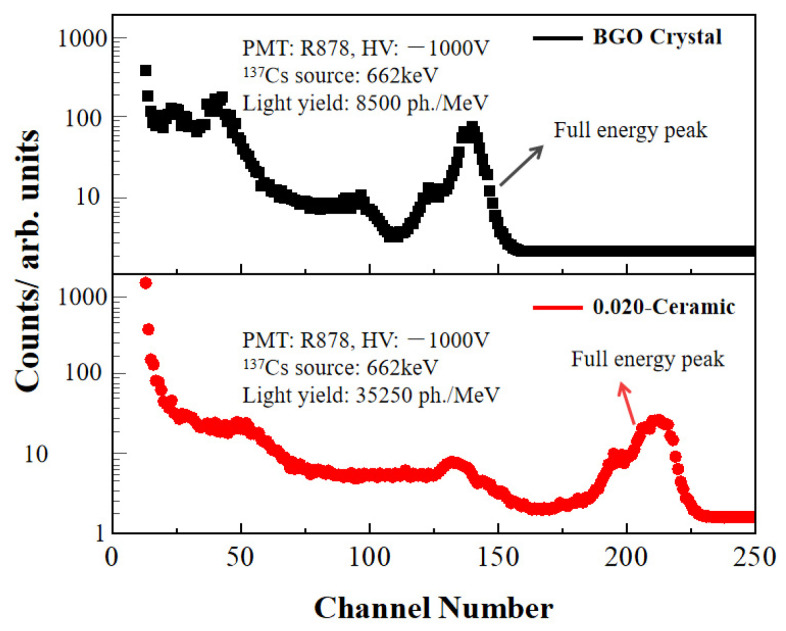
Pulse height spectrum acquired at 662 keV of 0.02 transparent ceramic and BGO single crystal.

Another important property for quantifying scintillation performance should be the scintillation light yield. State-of-the-art commercial scintillators for high-resolution PET systems are Bi_4_Ge_3_O_12_ (BGO), the multi-channel energy spectra of the as-prepared 0.02 transparent ceramic and a commercial BGO crystal with a size of 15 mm × 15 mm × 1 mm were recorded under 662 keV (^137^Cs) γ-rays exposure, as illustrated in Figure 14. The number of channels in the ceramic sample is 1.5 times more than that of BGO, indicating that its scintillation light yield is significantly higher than that of BGO. Based on the quantum efficiency conversion equation [40], the scintillation light yield of BGO is 8500 ± 550 ph/MeV and that of the 0.02 transparent ceramic is about 35,000 ± 1250 ph/MeV, demonstrating that the light yield of the as-fabricated Ce: GGAG transparent ceramic can be 3 times higher than BGO single crystals and about 1.5 times higher than the reported Ce: YAG scintillators [41]. 

A higher scintillation light yield would contribute to a better detector’s energy resolution and spatial resolution. However, scintillation properties of most of the previously reviewed highly transparent ceramics listed in Table 1 have not been evaluated, LED/LD lighting applications are particularly concerned instead. Table 2 summarizes the absolute light yields of several typical garnet ceramic scintillators. It can be concluded that the light yield is closely related to the component as well as the optical quality of the ceramic scintillators. The as-fabricated Ce: GGAG transparent ceramic shows remarkable light yield compared to the currently reported garnet transparent ceramics [35,41]. However, it is also worth noting that a GYGAG: Ce transparent ceramic with a light yield of 50,000 ph/MeV has been developed at Lawrence Livermore National Laboratory. This should be the highest light yield for the currently reported transparent ceramic scintillators. In addition, some other cases with high light yields were reported for gadolinium–gallium–aluminum garnet ceramics [33,39,42]. However, they were optically translucent or even opaque, which restricts their applications in many fields, such as high-sensitivity imaging. The enhancement of light yield should be ascribed to complicated factors, including the actual doping concentration of Ce^3+^ ions [43,44], energy migration due to the impurity phases, and light scattering near the detection layers of Si-APD [39]. The components of the gadolinium–gallium–aluminum garnet ceramics and the Ce-doping concentrations have not been elaborately optimized in the present work. Therefore, the optical quality and composition of the GGAG: Ce transparent ceramics must be comprehensively optimized to finally obtain higher quality Ce: GGAG transparent ceramics for scintillating applications.

## 4. Conclusions

In summary, transparent Ce-Doped Gd_3_(Al, Ga)_5_O_12_ (Ce: GGAG) ceramics have been successfully fabricated using nano-sized powders synthesized by the microwave-assisted homogenization precipitation (MAHP) method. The concentration of Ga ion is a crucial factor in achieving pure phase Ce: GGAG nanopowders and dense ceramics. A low cation concentration of 0.02 mol/L is advantageous for obtaining monodispersed Ce: GGAG powders and pre-sintered ceramics with high relative density. The Ce: GGAG transparent ceramic with a high in-line transmittance of 65.2% at 800 nm was obtained after further hot isostatically pressing processing. Additionally, the scintillation decay time was examined to be 35 ns, much lower than that of the Ce: GGAG single crystals. The light yield of the 0.02 ceramic was evaluated to be 35,000 ± 1250 ph/MeV, which was almost 3 times higher than that of a commercial Bi_4_Ge_3_O_12_ single crystal. The optical properties and scintillating characteristics demonstrate that the as-fabricated Ce: GGAG transparent ceramic has excellent potential for scintillating applications. 

## Figures and Tables

**Table 1:** Optical properties of the Gd_3_(Al, Ga)_5_O_12_ ceramics fabricated via different methods.

Composition	Fabrication Method	Sintering Aids	Pre-Sintering Atmosphere	HIP Parameter	Thickness	Highest Transmittance	Reference
Gd_3_Al_3_Ga_2_O_12_:Ce	Solid state reaction	MgO	Oxygen	NON	Information absence	~45%@545 nm	[33]
Gd_3_Al_2_Ga_3_O_12_:Ce	Solid state reaction	TEOS, MgO, CaO	Air	200 MPa 1500 °C	1.13 mm	50.1%@550 nm	[29]
Gd_3_Al_3_Ga_2_O_12_:Ce	Solid state reaction	Information absence	Oxygen	NON	1 mm	62%@558 nm	[34]
Gd_3_Al_3_Ga_2_O_12_:Ce	Solid state reaction	TEOS	Vacuum	NON	1.5 mm	~70@600 nm	[35]
Gd_3_Al_3_Ga_2_O_12_:Ce	Solid state reaction	ZrO_2_	Oxygen	NON	1 mm	73%@558 nm	[30]
Gd_3_Al_2_Ga_3_O_12_:Cr/Eu	Solid state reaction	Information absence	Vacuum	NON	1 mm	75.3%@800 nm	[36]
Gd_3_Al_3_Ga_2_O_12_:Ce	Solid state reaction	MgO	Oxygen	1500 °C	1 mm	78.6%@500–800 nm	[28]
Gd_3_(Al,Ga)_5_O_12_:Ce	Hot-pressing	NON	Vacuum	NON	1.8 mm	33%@550 nm	[37]
Gd_3_(Al,Ga)_5_O_12_:Ce	Ultrasonic chemical co-precipitation	Information absence	Oxygen	Yes	1 mm	51%@545 nm	[38]
Gd_3_(Al,Ga)_5_O_12_:Ce	Ultrasonic chemical co-precipitation	Information absence	Oxygen	Yes	1 mm	68%@545 nm	[38]
Gd_3_Al_3_Ga_2_O_12_:Ce	MAHP	NON	Oxygen	200 MPa 1480 °C	1 mm	65.2%@800 nm	This work

**Table 2:** Scintillation properties of typical ceramic scintillators.

Materials	Appearances	Light Yield/ph/MeV	Reference
Y_3_Al_5_O_12_:0.4%Ce	Transparent	24,600	[41]
Y_3_Al_2_Ga_3_O_12_:0.8%Ce	Transparent	13,700 ± 1400	[35]
Lu_3_Al_2_Ga_3_O_12_:0.8%Ce	Transparent	18,700 ± 1900	[35]
Gd_3_Al_2_Ga_3_O_12_:0.8%Ce	Transparent	15,500 ± 1600	[35]
GYGAG:Ce	Transparent	50,000	[19]
Gd_3_Al_3_Ga_2_O_12_:0.35%Ce	Translucent	31,500	[33]
Gd_3_(Al, Ga)_5_O_12_:Ce	Translucent	48,000	[42]
GAGG:1%Ce	Opaque	70,000	[39]
Gd3Al3Ga2O12:0.33%Ce	Transparent	35,000 ± 1250	This work

## Data Availability

Data are contained within the article.
